# Standardized Parenteral Nutrition for the Transition Phase in Preterm Infants: A Bag That Fits

**DOI:** 10.3390/nu10020170

**Published:** 2018-02-02

**Authors:** Ann-Marie Brennan, Mairead E. Kiely, Sarah Fenton, Brendan P. Murphy

**Affiliations:** 1 Department of Clinical Nutrition and Dietetics, Cork University Maternity Hospital, T12 YE02 Cork, Ireland; 2 Irish Centre for Fetal and Neonatal Translational Research (INFANT), University College Cork, T12 DFK4 Cork, Ireland; m.kiely@ucc.ie (M.E.K.); sarah.fenton@hse.ie (S.F.); brendanpaul.murphy@hse.ie (B.P.M.); 3 Cork Centre for Vitamin D and Nutrition Research, School of Food and Nutritional Sciences, University College Cork, T12 Y337 Cork, Ireland; 4 Pharmacy Department, Cork University Hospital, T12 YE02 Cork, Ireland; 5 Department of Neonatology, Cork University Maternity Hospital, T12 YE02 Cork, Ireland

**Keywords:** standardized parenteral nutrition, transition phase, nutrient modeling, amino acids, enteral nutrition, preterm infants

## Abstract

The optimal composition of standardized parenteral nutrition (SPN) is not yet known, contributing to nutrient deficit accrual and growth failure, with the period of parenteral nutrition weaning, i.e., transition (TN) phase, being identified as particularly vulnerable. We created a comprehensive nutrition database, representative of the nutritional course of a diverse range of preterm infants (*n* = 59, birth weight ≤ 1500 g, gestation < 34 weeks) by collecting hourly macronutrient intake data as part of a prospective, observational study over 19 months. Using a nutrient modeling technique for the TN phase, various amino acid (AA) concentrations of SPN were tested within the database, whilst acknowledging the nutritional contribution from enteral feeds until target AA intakes were consistently achieved. From the modeling, the AA composition of SPN was determined at 3.5 g/100 mL, which was the maximum to avoid exceeding target intakes at any point in the TN phase. However, in order to consistently achieve target AA intakes, additional nutritional strategies were required, which included increasing the exclusion of enteral feeds in fluid and nutrient calculations from <20 mL/kg/day to <40 mL/kg/day, and earlier fortification of breastmilk at 80 mL/kg/day. This data-driven nutrient modeling process supported the development of an improved SPN regimen for our preterm population in the TN phase.

## 1. Introduction

The nutrition course of the preterm infant has more recently been described as three discrete nutrition phases, i.e., the parenteral nutrition (PN) phase when the infant in entirely dependent on PN for nutrition, the enteral nutrition (EN) phase when the infant is fully established on milk feeds, and the transition (TN) phase [1,2,3] when PN is being weaned with advancing enteral feeds. The TN phase is an extremely complex nutritional period characterized by multiple nutrient sources (PN and EN), and has been reported to last from 7 [1] to 10 days [2]. Despite some studies showing that early, high amino acid (AA) intakes were associated with improvements in growth, glycaemic tolerance and electrolyte homeostasis [4], the TN phase has recently been exposed as a period of cumulative nutrient deficits, in particular AA [1], and compromised growth [3]. It has been reported that infants who experience growth failure in the TN phase are five times more likely to be discharged with a weight < 10th centile for corrected gestational age (GA) [2]. Currently, there is a lack of specific nutrition recommendations for the TN phase. International recommended nutrient intakes (RI) are based on the infant being either solely on PN [5,6,7] or EN [5,6,8] with no specific guidelines in relation to weaning PN, whilst minimizing the disruption to nutrient delivery during the incremental introduction of enteral feeds [1]. We recently proposed that the lack of specific TN phase nutrition recommendations weakens the nutritional management of the phase, and may be directly contributing to the accumulation of nutrient deficits [1].

Miller and colleagues have recently shown improved nutrient intakes, in particular AA, and growth in the TN phase [2]. This was achieved through the use of concentrated PN, and took into consideration the nutritional contribution from enteral feeds. However, target AA intakes were not consistently achieved throughout the phase. Therefore, the optimal composition of PN to achieve target AA intakes for the TN phase is not yet known. We reported from a prospective observational study, substantial nutrient deficits, in particular AA, throughout the entire TN phase, despite the implementation of commonly used nutritional strategies, i.e., ‘mL per mL’ PN weaning protocol, and fortification of breastmilk (BM) [1]. There is increasing evidence to support the use of concentrated standardised PN (SPN) regimens [9,10]. However, increasing the AA concentration of PN should take the increasing nutritional contribution from enteral feeds into consideration throughout the TN phase, as recent observations from early enhanced nutritional management, i.e., high AA intake, have reported unintended adverse effects [11,12,13,14,15].

Dietary modeling techniques are useful in translating RI into practical nutrition guidelines to achieve target intakes in older populations [16,17,18,19,20]. In these studies, dietary modeling was based on population nutrient intake databases; however, in the neonatal setting, such databases do not exist and dietary modeling techniques have not been exploited. In this study, we describe a novel process to determine the optimal AA composition of SPN for the TN phase using nutrient modeling of a preterm nutrition database based on actual nutrient intake data [21]. To overcome the lack of international guidelines for this phase, we used our recently described application of current international RI, based on the infants’ ability to tolerate enteral feeds, and the dominant nutrition source [1] to define target AA intakes, throughout the TN phase.

## 2. Materials and Methods

### 2.1. Study Design and Population

As part of a prospective, observational, longitudinal study on nutrition and growth in 59 preterm infants (birth weight (BW) ≤ 1500 g and GA < 34 weeks), we constructed a comprehensive nutrition database based on actual hourly nutrient intake data [21]. The study took place at Cork University Maternity Hospital neonatal unit (NU) over a 19-month period from March 2010 to October 2011; the study commenced after updating our nutrition guidelines (supplementary Table S1) on the basis of recent international recommendations [6,7,8]. Written parental informed consent for participation was obtained. Exclusion criteria included congenital or chromosomal abnormalities and use of postnatal steroids for neonatal chronic lung disease. The Cork University Hospitals Clinical Research Ethics Committee approved the study protocol (reference no. ECM 4 (e)) and the study was registered at ClinicalTrials.gov (NCT01881256).

Nutrient data was stratified by infant BW to allow precise comparisons with international nutrition recommendations [5,6], which are primarily BW based. We defined infants born < 1000 g as extremely low birth weight (ELBW, *n* = 12) and infants born between 1000 and 1500 g as very low birth weight (VLBW). We subdivided VLBW infants by GA to distinguish between early (VLBW < 30 weeks (*n* = 23)) versus late preterm (VLBW ≥ 30 weeks (*n* = 24)), as those born < 30 weeks GA are considered to be at increased nutritional risk, due to their more immature physiological development [22].

### 2.2. Nutrition Database Design and Construction

The nutrition database was constructed in MS Excel (Microsoft, Redmond, WA, USA). Total daily nutrient intakes were recorded retrospectively from PN prescriptions, drug prescriptions and daily fluid balance sheets, which were documented hourly enabling observed (not prescribed) parenteral and enteral intakes to be precisely determined for each infant on a 24-h basis. A ‘parenteral and enteral intake’ spreadsheet was created to record the intake, in terms of volume per kg, for each infant, for each nutritional source by the investigator (AMB). Day of life was defined from hour of birth, i.e., day 1 of life was the first 24-h period. Use of hourly intake source data captured all changes to the parenteral and enteral prescriptions administered to the infants each day.

A ‘nutrient composition’ spreadsheet was created with composition data for each parenteral, i.e., intravenous (IV) dextrose 10%, 12.5%, PN, and enteral solution, i.e., breastmilk (BM), fortified BM and preterm formula. This was constructed with the most accurate nutrient composition data at that time according to product specifications. BM nutrient provision was estimated based on published values for transitional BM at 1.5 g protein/100 mL [23,24]. In the absence of definitive evidence of the bioavailability data for enteral nutrients in preterm infants, 100% bioavailability was assumed in line with current recommendations [23]. Energy calculations for PN were glucose = 3.4 kcal/g [25], AA = 3.7 kcal/g (Vaminolact; Fresenius Kabi, Graz, Austria), and lipid = 10 kcal/g (Intralipid 20% and SMOF; Fresenius Kabi, Graz, Austria). Energy values for EN products were according to most up-to-date product specifications which were based on 4 kcal/g for carbohydrate, 4 kcal/g for protein and 9 kcal/g for fat [26], and based on published values for transitional BM at 67 kcal/100 mL [24]. For individualized PN prescriptions, individual nutrient intakes contained within each infusion were individually calculated. The ‘parenteral and enteral intake’ and ‘nutrient composition’ spreadsheets were linked to enable calculation of observed macronutrient and energy intakes for both, which were summed to provide total daily intakes, an approach previously described for the TN phase [1,2]. The database was designed such that any update to the individual spreadsheets would recalculate the intake data.

### 2.3. Nutrition Database Modeling for the TN Phase

#### 2.3.1. TN Phase Classification

Prior to nutrient modeling, once the database, which followed the infant’s chronological age, was completed, daily nutrient intakes were analyzed according to enteral feed volumes (EFV), to allow classification according to the TN phase, previously described elsewhere [1]. The TN phase was defined as EFV greater or equal to 20 mL/kg/day but less than 120 mL/kg/day (PN was generally discontinued once EFV reached 120 mL/kg/day as enteral feeds provided adequate protein intakes, i.e., 3.5 g/kg/day at this EFV). We subcategorized the TN phase into a PN-dominant TN phase (defined by EFV < 80 mL/kg/day, i.e., approximately 50% of full enteral feeds), and an EN-dominant TN phase (defined by EFV ≥ 80 mL/kg/day). We grouped EFV per 10 mL aliquots, i.e., 20 mL/kg/day represents 20 to 29 mL/kg/day, and mapped daily AA/protein intakes to the corresponding EFV received on that day by each infant.

#### 2.3.2. Fluid and Nutritional Constraints

In nutrient modeling, the outcome of each decision (e.g., PN composition, or other nutritional strategies) must be constrained by a minimum and maximum range of limits or targets, i.e., PN or EN RI [16]. These limits or targets are termed constraints (Table 1). Target AA intakes were set according to the recently described TN phase RI [1]. During the PN-dominant TN phase (EFV < 80 mL/kg/day), target AA intakes were based on PN RI [6] and during the EN-dominant TN phase (EFV ≥ 80 mL/kg/day), target AA intakes were based on EN RI [5]; this approach was used to account for the differences in bioavailability between parenteral and enteral nutrient sources [1]. For ease of comparison and improved accuracy between AA/protein intakes and target intakes, during the PN-dominant TN phase, enteral protein intakes were converted to the corresponding AA equivalent using the following equation: 1 g protein = 1.13 g AA [27] and during the EN-dominant TN phase, parenteral AA were converted to the corresponding protein equivalent using the following equation: 1 g AA = 0.89 g protein. 

Actual daily total IV fluid, i.e., parenteral (aqueous and lipid PN, and any IV dextrose), and enteral feeds were set as non-modifiable fluid constraints, in order to reflect the clinical realities of fluid management, and challenges with enteral feed tolerance in this population. However, the ratio of the different components of the infant’s IV fluid intake could be modified within reasonable clinical parameters.

#### 2.3.3. Nutrient Modeling Steps

**Figure 1:**
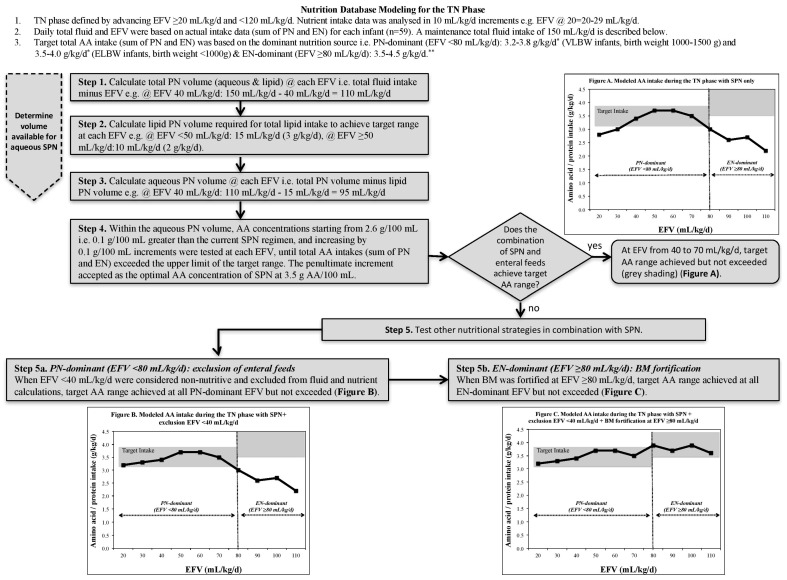
Flow chart of nutrient modeling steps. TN: transition; EFV: enteral feed volume; PN: parenteral nutrition; EN: enteral nutrition; AA: amino acid; SPN: standardized parenteral nutrition; BM: breastmilk. * PN recommendations [6]. ** EN recommendations [5].

Using the linked design of the ‘parenteral and enteral intake’ with the ‘nutrient composition’ spreadsheets, and using a manual nutrient modeling technique [16], the investigator AMB, an experienced neonatal dietitian, manipulated the spreadsheets in a clinically relevant sequential manner, as described in Figure 1. The volume available for aqueous SPN had to be determined first (Figure 1, Steps 1–3). After these steps, modeled AA intakes were reviewed to assess if target AA intakes were achieved but not exceeded at each EFV, defined by the calculated sum totals of daily parenteral and enteral intakes being within target ranges. Additional modeling steps were undertaken in combination with SPN, and included the testing of various nutritional strategies, i.e., exclusion of enteral feeds from fluid and nutrient calculations (Figure 1, Step 5a), and fortification of BM (Figure 1, Step 5b) until target AA intakes were consistently achieved throughout the TN phase. In addition to AA, the provision of lipid, carbohydrate and energy were also considered to ensure optimal macronutrient compostion of SPN for the TN phase.

### 2.4. Statistics

Statistical analysis was conducted using PASW Statistics Version 20.0 (SPSS, IBM, Armonk, NY, USA). Descriptive statistics (mean (+/−SD, SEM) and prevalence data where appropriate) were determined for all variables. Characteristics of subjects in the three groups were compared using either one-factor analysis of a variance (ANOVA) followed by Tukey’s test (parametric data) for continuous variables or a chi-square test for categorical variables. A *P* value of <0.05 was considered statistically significant.

## 3. Results

The parents of 74 eligible infants were invited to participate and 70 provided written informed consent, of whom 2 subsequently died, 4 were transferred to another NU, 2 were withdrawn by parents and 3 were excluded as they could not complete the protocol due to clinical complications. Thus, the final sample size was 59 infants; 24 were boys, 12 were ELBW infants and 57 were Caucasian. Clinical and nutritional baseline data are summarized in Table 2.

**Figure 2:**
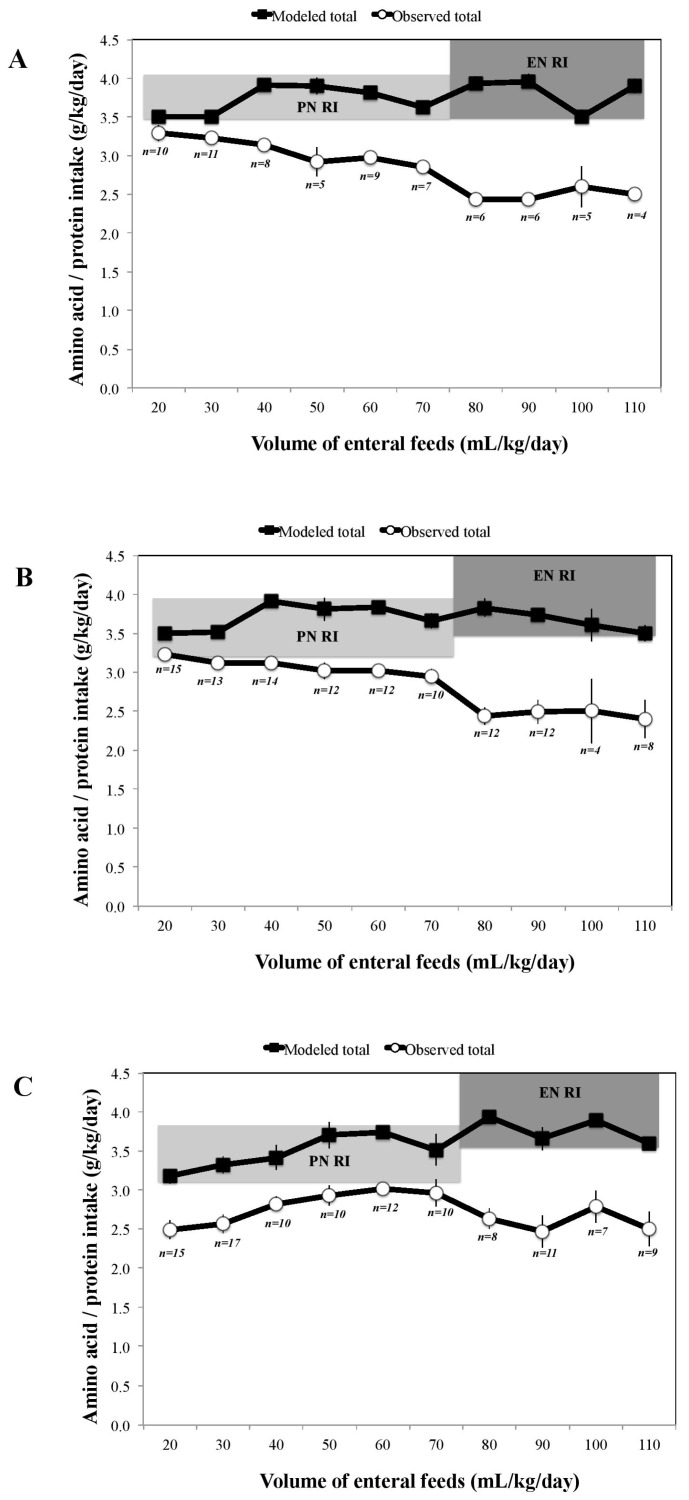
Mean (SEM) modeled versus observed total (sum of parenteral and enteral) amino acid/protein intakes during the transition (TN) phase (previously defined as enteral feeds ≥ 20 and <120 mL/kg/day) in ELBW (**A**); VLBW < 30 weeks (**B**) and VLBW ≥ 30 weeks (**C**) infants, compared with parenteral nutrition (PN) recommended intakes (RI) [6] during the PN-dominant TN phase (enteral feeds < 80 mL/kg/day; light grey banding), and enteral nutrition (EN) RI [5] during the EN-dominant TN phase (enteral feeds ≥ 80 mL/kg/day; dark grey banding).

From the modeling, the optimal AA composition of SPN was determined at 3.5 g/100 mL (previously 2.5 g/100 mL), as shown in Figure 1, Step 4. This was the maximum concentration possible to avoid exceeding target AA intakes at any point in the TN phase. However, in order to achieve target AA intakes at all EFV, modifications to other nutritional strategies were required. During the PN-dominant TN phase, at EFV 20 and 30 mL/kg/day, it was necessary to increase the exclusion of enteral feeds in fluid and nutrient calculations from <20 mL/kg/day to <40 mL/kg/day (Figure 1, Step 5a). This modification resulted in redefining the beginning of the TN phase to 40 mL/kg/day instead of previously at 20 mL/kg/day. During the EN-dominant TN phase, earlier fortification of BM at 80 mL/kg/day instead of previously at 120 mL/kg/day was required (Figure 1, Step 5b). Figure 2 illustrates how the combined application of these three nutritional strategies (modeled total) resulted in the consistent achievement of target AA intakes across the three infant groups when compared with our observational intake data (observed total).

The achievements of target intakes for lipid, carbohydrate, and energy for the TN phase are shown in supplementary Figures S1–S3. In addition, the clinical application of these nutrient modeling outputs is summarized in the integrated TN phase protocol described in Table 3.

## 4. Discussion

In this study, we established, through nutrient modeling of a comprehensive preterm nutrition database, the optimal AA composition of SPN for the TN phase. Our analysis showed that the achievement of target AA intakes required not just an increased AA concentration of our SPN bag, but also modifications to other nutritional strategies, which included exclusion of some EFV from fluid and nutrient calculations and the earlier fortification of BM. In this study, the nutrition database was representative of the preterm infant’s actual nutritional course, overcoming the well documented discrepancy between prescribed versus actual intakes [28,29] related to the clinical realities facing preterm infants day to day, and represented a broad range of preterm infants, i.e., ELBW and VLBW infants. In the literature, nutrient modeling techniques have been shown to be useful in translating RI into practical nutrition guidelines to achieve target nutrient intakes in other populations [16,17,18,19,20]. To the best of our knowledge, this is the first time that nutrient modeling of a preterm nutrition database has been used to determine the composition of SPN to improve the nutritional management of preterm infants.

It has been proposed that in order to optimize the nutritional management of the TN phase, consideration of both PN and EN sources is required [1,2]. However, recent efforts to apply this approach in practice have not yet yielded the consistent achievement of target AA intakes [2]. In our current analysis, the nutrition database, when used as a test environment, facilitated a more detailed consideration of the relationship between the multiple nutrient sources that characterize the TN phase, than has previously been described. We tested a range of AA concentrations for PN; similar to those reported in the literature, i.e., 3.8 g/100 mL [30] and 4.2 g/100 mL [10], and observed that target intakes were exceeded at some EFV once the nutritional contribution from enteral feeds was considered, in our population. Similarly, when we tested the BM fortification as early as 50 mL/kg/day, as has been recently suggested in the literature [31], target AA intakes were exceeded. Our standard practice of including EFV in fluid and nutrient calculations from as early as 20 mL/kg/day contributed to suboptimal AA intakes amongst some VLBW infants but exclusion of higher volumes, i.e., ≥40 mL/kg/day would have led to excessive AA intakes. Our data analysis supports the recent recommendation of excluding enteral feeds < 40 mL/kg/day in fluid calculations [4], and identifying this as the optimal EFV to begin weaning PN, i.e., start of TN phase. Nutrient modeling ensured nutritional strategies, i.e., PN concentration and BM fortification worked together in a complementary manner to support the achievement of target AA intakes, without exposing infants to excessive intakes, and may support the development and refinement of integrated TN phase protocols. The modeling outputs and resultant TN phase protocol reflect our fluid and nutritional constraints but the principles of this process can be universally applied. Additionally, it should be noted that fluid and nutritional constraints are not fixed due to the advancing evidence-base and changes to nutritional products.

There is evidence that a TN phase protocol incorporating concentrated PN is a simple, practical, and effective method to help address nutrient deficits inherent in the TN phase of preterm infants, and results in improved growth [2]. Despite the knowledge of early nutrient deficit accrual [1,32], due to the recent reports of safety concerns regarding early enhanced nutritional management [11,12,13,14,15], we were justifiably reluctant to modify our PN composition. We used nutrient modeling to provide a scientific approach to determine optimal SPN composition, and develop an integrated TN phase protocol, thus allowing a novel data-driven rather than best-guess rationale for making changes. This provided confidence and reassurance to the clinical multidisciplinary team that the proposed changes in unit practice, i.e., PN composition and other nutritional strategies, would be safe and effective in our diverse preterm population. We believe that this nutrient modeling process provides a platform to support change in clinical practice and could inform future development and composition of nutritional products, i.e., SPN bags, BM fortifiers and preterm formulas.

A strength of this study is the consistent involvement of a neonatal dietitian who was responsible for constructing and manually modeling the nutrition database. Additionally, testing across the three preterm infant groups ensured that a single SPN composition within an integrated TN phase protocol could meet the needs of our diverse preterm population. A limitation of the study was that the protein content of BM was not analyzed, and published values [23,24] were used to calculate nutrient intakes. Therefore, modeled and observed protein intakes relied on estimated rather than the actual content of BM. However, the value we used for transitional BM of 1.5 g protein/100 mL is consistent with the recent recommendation from Cormack and colleagues, 2016 [23]. Future studies evaluating the impact of nutrient intakes in preterm infants should use analyzed BM values rather than estimated values. Another limitation in the field of neonatal nutrition is the lack of definitive evidence of the bioavailability of enteral nutrients. In the future, we would welcome the establishment of bioavailability data for this population to facilitate the appropriate analysis of nutrient intakes from multiple sources, i.e., during the TN phase.

## 5. Conclusions

The transition phase is a nutritionally complex and vulnerable period in preterm infants where the achievement of target nutrient intakes is challenging. We have demonstrated that nutrient modeling is a valuable process to determine the amino acid composition of standardized parenteral nutrition, and modifications to other nutritional strategies within an integrated TN phase protocol that support optimal amino acid intakes during the transition phase. Data derived from nutrient modeling provides evidence and a scientific basis to support nutritional change management in neonatal units.

## Figures and Tables

**Table 1:** List of local nutritional and fluid constraints used in nutrient modeling of the preterm nutrition database for the TN phase.

Nutritional *	Amino acid, g/kg/day	PN-dominant TN phase: 3.5–4.0 (ELBW), 3.2–3.8 (VLBW) EN-dominant TN phase: 3.5–4.5 **
	Lipid, g/kg/day	PN-dominant TN phase: 3.0–4.0 *** EN-dominant TN phase: 4.8–6.6
	Glucose, g/kg/day	PN-dominant TN phase: 13.0–17.0 (ELBW), 9.7–15.0 (VLBW) EN-dominant TN phase: 11.6–13.2
	Energy, kcal/kg/day	PN-dominant TN phase: 90–115 EN-dominant TN phase: 110–130
Fluid	Total daily fluid intake	Actual total daily fluid intakes were not altered
	Enteral feed intake	Actual daily enteral feed intakes were not altered
	Parenteral lipid	Each g lipid is delivered in 5 mL (20% concentration)
	IV fluid concentration	PN regimens and IV fluids set at a maximum 12.5% dextrose to allow flexibility for peripheral and central access

ELBW: extremely low birth weight infant; IV: intravenous; PN: parenteral nutrition; TN: transition; VLBW: very low birth weight infant. * During the PN-dominant TN phase, when nutrition intake was predominantly parenterally sourced (defined by enteral feeds < 80 mL/kg/day), nutritional constraints were based on Tsang’s PN ‘growing’ recommendations [6]. During the EN-dominant TN phase, when nutrition intake was predominantly enterally sourced (defined by enteral feeds ≥ 80 mL/kg/day), nutritional constraints were based on the most up-to-date EN recommendations [5]. ** Represents enteral protein recommendations. *** In the PN-dominant TN phase, an initial upper lipid constraint of 4.0 g/kg/day was assigned. However, in order to achieve target energy intakes whilst respecting all other constraints, the upper constraint was increased to 4.8 g/kg/day (combined parenteral and enteral intake) with a maximum of 3 g/kg/day from parenteral lipid. This approach took into account the reduced bioavailability of enteral lipid when compared to a PN recommendation.

**Table 2:** Baseline population characteristics and observed nutrient intake data of 59 preterm infants.

	ELBW (*n* = 12)	VLBW < 30 Weeks (*n* = 23)	VLBW ≥ 30 Weeks (*n* = 24)	*P* *
Perinatal and postnatal data				
Male	5 (42%)	9 (39%)	10 (42%)	0.98
Gestational age, weeks	26.9 ± 1.8 ^a^	28.0 ± 0.8 ^b^	31.3 ± 1.1 ^c^	<0.001
Birth weight, g	834 ± 113 ^a^	1220 ± 120 ^b^	1330 ± 140 ^c^	<0.001
SGA at birth	4 (33%)	1 (4%)	11 (46%)	0.005
Maternal hypertension	3 (25%)	1 (4%)	7 (29%)	0.08
Cesarean section	7 (58%)	14 (61%)	21 (88%)	0.07
Antenatal steroids	11 (92%)	20 (87%)	22 (92%)	0.84
Multiple births	6 (50%)	11 (48%)	16 (67%)	0.39
Nasal CPAP	12 (100%)	22 (96%)	17 (71%)	0.01
Conventional ventilation after birth	9 (75%)	13 (57%)	4 (17%)	0.001
Chronic lung disease	3 (25%)	1 (4%)	0	0.02
Patent ductus arteriosus	9 (75%)	12 (52%)	6 (25%)	0.01
Late onset sepsis	2 (17%)	5 (22%)	2 (8%)	0.44
Nutrition data				
Age PN commenced, day	1.0 ± 0.0	1.1 ± 0.3	1.2 ± 0.4	0.24
Age lipid commenced, day	1.5 ± 0.5 ^a,b^	2.2 ± 1.1 ^a^	1.6 ± 1.0 ^b^	0.04
Individualized PN	12 (100%)	16 (70%)	3 (13%)	<0.001
Duration of PN phase, day	6.3 ± 2.8 ^a^	4.5 ± 1.7 ^b^	2.6 ± 1.1 ^c^	<0.001
Duration of TN phase, day	9.0 ± 2.2 ^a^	6.0 ± 3.0 ^b^	5.9 ± 3.0 ^b^	0.005
Days receiving PN	15.3 ± 3.5 ^a^	10.5 ± 3.7 ^b^	8.5 ± 3.1 ^b^	<0.001
Age EN commenced, day	2.9 ± 2.0 ^a^	2.9 ± 0.6 ^a^	1.9 ± 0.7 ^b^	0.003
Age when feeds ≥ 150 mL/kg/day achieved, day	17.7 ± 4.5 ^a^	13.0 ± 4.0 ^b^	10.7 ± 2.7 ^b^	<0.001
Fortification of BM at EN volume, mL/kg/day	117 ± 22	121 ± 20	125 ± 20	0.61
BM, any **	12 (100%)	23 (100%)	21 (88%)	0.10
BM, >80% of total enteral feeds	12 (100%)	21 (91%)	19 (79%)	0.16

Data are presented as number (percentage) and mean ± SD. BM: breastmilk; CPAP: continuous positive airway pressure; EN: enteral nutrition; PN: parenteral nutrition; SGA: small for gestational age (birth weight < 10th percentile); TN: transition. * One-factor ANOVA followed by Tukey’s test or chi-square test for percentages. Superscript letters denote significant differences between groups, *P* < 0.05. ** A total of four infants received donor BM (range 2–20 days).

**Table 3:** A proposed integrated TN phase protocol.

Enteral Nutrition	Parenteral Nutrition			Nutritional Strategy
Enteral Feed Volume mL/kg/day	Target Aqueous Volume mL/kg/day	Target Lipid Volume mL/kg/day	Target Total PN Volume mL/kg/day	
40	95	15 (3 g/kg/day)	110	
50	85	15 (3 g/kg/day)	100	
60	80	10 (2 g/kg/day)	90	Reduce lipid from 3 to 2 g/kg/day
70	70	10 (2 g/kg/day)	80	
80	60	10 (2 g/kg/day)	70	Commence breastmilk fortifier
90	50	10 (2 g/kg/day)	60	
100	40	10 (2 g/kg/day)	50	
110	30	10 (2 g/kg/day)	40	
120	Consider stopping PN			

TN: transition; PN: parenteral nutrition.

## Supplementary Materials

The following are available online at http://www.mdpi.com/2072-6643/10/2/170/s1, Figure S1: Mean (SEM) modeled versus observed total (sum of parenteral and enteral) lipid intakes during the transition (TN) phase (previously defined as enteral feeds ≥ 20 and <120 mL/kg/day) in ELBW (A), VLBW < 30 weeks (B) and VLBW ≥ 30 weeks (C) infants, compared with parenteral nutrition (PN) recommended intakes (RI) [6] during the PN-dominant TN phase (enteral feeds < 80 mL/kg/day; light grey banding), and enteral nutrition (EN) RI [5] during the EN-dominant TN phase (enteral feeds ≥ 80 mL/kg/day; dark grey banding). Figure S2: Mean (SEM) modeled versus observed total (sum of parenteral and enteral) carbohydrate intakes during the transition (TN) phase (previously defined as enteral feeds ≥ 20 and <120 mL/kg/day) in ELBW (A), VLBW < 30 weeks (B) and VLBW ≥ 30 weeks (C) infants, compared with parenteral nutrition (PN) recommended intakes (RI) [6] during the PN-dominant TN phase (enteral feeds < 80 mL/kg/day; light grey banding), and enteral nutrition (EN) RI [5] during the EN-dominant TN phase (enteral feeds ≥ 80 mL/kg/day; dark grey banding). Figure S3: Mean (SEM) modeled versus observed total (sum of parenteral and enteral) energy intakes during the transition (TN) phase (previously defined as enteral feeds ≥ 20 and <120 mL/kg/day) in ELBW (A), VLBW < 30 weeks (B) and VLBW ≥ 30 weeks (C) infants, compared with parenteral nutrition (PN) recommended intakes (RI) [6] during the PN-dominant TN phase (enteral feeds < 80 mL/kg/day; light grey banding), and enteral nutrition (EN) RI [5] during the EN-dominant TN phase (enteral feeds ≥ 80 mL/kg/day; dark grey banding). Table S1: description of the nutrition guideline at the Cork University Maternity Hospital neonatal unit at the time of the study.
