# Supplementary material for: Standardized Parenteral Nutrition for the Transition Phase in Preterm Infants: A Bag That Fits
Source: Nutrients. 2018 Feb 2;10(2):170. doi: 10.3390/nu10020170 (PMC5852746; doi:10.3390/nu10020170)
Supplement: Supplementary file 1 [file nutrients-10-00170-s001.doc]

**Figure S1.** Mean (SEM) modeled vs. observed total (sum of parenteral and enteral) lipid intakes during the transition (TN) phase (previously defined as enteral feeds ≥20 and <120 mL/kg/d) in ELBW (A), VLBW <30 weeks (B) and VLBW ≥30 weeks (C) infants, compared with parenteral nutrition (PN) recommended intakes (RI) [[6]](#_ENREF_6) during the PN-dominant TN phase (enteral feeds <80 mL/kg/d; light grey banding), and enteral nutrition (EN) RI [5] during the EN-dominant TN phase (enteral feeds ≥80 mL/kg/d; dark grey banding).

A

B

C

**Figure S2.** Mean (SEM) modeled vs. observed total (sum of parenteral and enteral) carbohydrate intakes during the transition (TN) phase (previously defined as enteral feeds ≥20 and <120 mL/kg/d) in ELBW (A), VLBW <30 weeks (B) and VLBW ≥30 weeks (C) infants, compared with parenteral nutrition (PN) recommended intakes (RI) [[6]](#_ENREF_6) during the PN-dominant TN phase (enteral feeds <80 mL/kg/d; light grey banding), and enteral nutrition (EN) RI [5] during the EN-dominant TN phase (enteral feeds ≥80 mL/kg/d; dark grey banding).

A

B

C

**Figure S3.** Mean (SEM) modeled vs. observed total (sum of parenteral and enteral) energy intakes during the transition (TN) phase (previously defined as enteral feeds ≥20 and <120 mL/kg/d) in ELBW (A), VLBW <30 weeks (B) and VLBW ≥30 weeks (C) infants, compared with parenteral nutrition (PN) recommended intakes (RI) [[6]](#_ENREF_6) during the PN-dominant TN phase (enteral feeds <80 mL/kg/d; light grey banding), and enteral nutrition (EN) RI [5] during the EN-dominant TN phase (enteral feeds ≥80 mL/kg/d; dark grey banding).

A

B

C

**Table S1.** Description of the nutrition guideline at the Cork University Maternity Hospital neonatal unit at the time of the study

SPN=standardised parenteral nutrition; IPN=individualised parenteral nutrition.

| Fluid | Day 1 of life: commenced dextrose 10% at 60-80 mL/kg/d.  Increased by daily increments of 20 mL/kg/d to maintenance of 150 mL/kg/d. |
| --- | --- |
| PN (aqueous) | Day 1 of life: dextrose 10% changed to *starter* SPN (SPN1) within 12 hours of birth.  Day 2 of life onwards: infants <1000 g prescribed IPN & infants 1000-1500 g prescribed *follow-on* SPN (SPN2).  IPN changed to SPN2 when feeds reached 80 mL/kg/d approximately.  PN discontinued when enteral feeds reach 100-120 mL/kg/d and infant clinically and biochemically stable. |
| Amino Acid | 2.5 g/kg/d from day 1.  Increased by daily increments of 0.5 g/kg/d to a maximum of 3.5 g/kg/d. |
| Lipid | 1 g/kg/d from day 1.  Increased by daily increments 0.5 g/kg/d to a maximum of 3 g/kg/d. |
| Glucose | 6-8 g/kg/d (4.2-5.6 mg/kg/min) from day 1.  Increased by daily increments 2 g/kg/d to a maximum of 15 g/kg/d. |
| Enteral feeds | Commenced within 24-72 hours after birth when clinically stable and human milk is available at 10-30 mL/kg/d, depending on birth weight and clinical condition.  Increased by daily increments of 10-30 mL/kg/d depending on birth weight and feed tolerance.  Enteral feed ≥20 mL/kg/d were included as part of the total fluid and nutrient calculations. As enteral feeds advanced, sequential reductions in aqueous and lipid infusion rates were made to maintain a total fluid intake at 150 mL/kg/d approximately.* |
| Choice of milk | Breastmilk or preterm formula.  Full fortification of breastmilk when enteral feeds reached 100 mL/kg/d. |
| Enteral feed volumes | 160-180 mL/kg/d fortified breastmilk.  160 mL/kg/d preterm formula. |

Day 1 of life: defined as the first 24 hours after birth.

SPN1: A low electrolyte *starter* SPN regimen designed for preterm infants from birth provided 2.2 g amino acids and 42 kcal or 3.1 g amino acids and 46 kcal per 100 mL [the amino acid composition was increased after the enrollment of 40 infants due to product reformulation by company) (Vaminolact, Fresenius Kabi, Graz, Austria)].

SPN2: A *follow-on* SPN regimen suitable from day of life 2 onwards provided 2.5 g amino acids and 43 kcal per 100 mL.

Preterm formula (2.5 g protein and 80 kcal per 100 mL, Cow and Gate Nutriprem 1, Bledina, Steenvoorde, France). Breastmilk fortifier (Cow & Gate Nutriprem, Nutricia, Cuijk, The Netherlands) contained 0.8 g protein and 16 kcal added to 100 mL breastmilk.

*The PN weaning strategy was based on a “mL per mL” titration, where both the aqueous and lipid phases were proportionately reduced as enteral feeds increased, to maintain a total fluid intake of 150 mL/kg/d approximately. No adjustment was made to the PN volume ordered or the nutrient concentration in the PN fluids as enteral feeds advanced.
